# Gulnaz Azhymambetova: leading change in nursing care

**DOI:** 10.2471/BLT.22.030722

**Published:** 2022-07-01

## Abstract

Andrei Shukshin talks to Gulnaz Azhymambetova about empowering nurses to deliver better patient care in Kyrgyzstan.

*Q: When did you decide that you wanted to be a nurse?*

A: In fact, growing up I wasn’t sure if I wanted to be a teacher or a nurse, but I knew I wanted to be one or the other and could never imagine myself doing anything else. I have very positive early memories of the family nurse that came into our home. I was born in the village of Byzak in the Tian Shan mountains and, in the 1990s, there were no doctors in our area. So, our family was looked after by this nurse and I had an image of this kind, caring and respected person who was always ready to help. I also had a cousin who was a medical officer and I always looked up to him with admiration. After graduating high school, I passed the exams for both the teaching and medical universities in Bishkek. I made my final decision when the teachers in the Faculty of Higher Nursing Education at the Medical Academy told me that I would be able to realize both of my ambitions, training as a nurse but also developing teaching and management skills at the same time. I studied for five years and graduated as a nursing management specialist and professor in 2004.

*Q: So, did you teach or practice?*

A: Straight after graduating, I got a job as part of a family doctor group at a medical centre in Bishkek. Family doctor groups, which are usually comprised of a doctor and a nurse, are a core part of primary health care provision in Kyrgyzstan and are attached to family medical centres that operate in cities and towns. In rural areas we have general practice centres that combine a family medicine centre and a hospital, as well as an obstetric facility, where prenatal and perinatal primary health-care services are provided only by nurses. Family nurses also play an important role in the prevention, monitoring and care of diabetes and hypertension. We are also responsible for the delivery of immunization and were in the front line of COVID-19 (coronavirus disease 2019) health service delivery including vaccination.

*Q: How hard has the pandemic been for nurses in Kyrgyzstan?*

A: When the pandemic broke, the workload nurses had to shoulder in hospitals was truly incredible, with some of them having to care for as many as 40 patients each – taking nose and mouth swabs, monitoring patients, measuring temperature, blood oxygen saturation, assisting with intubations and ventilators, assisting patients, many of whom were in respiratory distress, and all that wearing protective gear. Many nurses contracted COVID-19 themselves. The psychological impact was enormous and there was a lot of burn-out. The pandemic also revealed significant weaknesses in our nursing practices.

“We need to transform nurses from simple assistants.”

*Q: In what way?*

A: Well for one thing it became apparent how rigid our nursing was. Since the days of the Soviet Union, nurses have tended to be assigned specific functions here. So, there would be certain nurses who did wound-dressing, certain nurses who did procedures or injections, etc. During the pandemic one of the main tasks nurses were called upon to perform was intravenous injection of medication and it turned out that nurses who had been doing dressings for thirty years had a hard time hitting the vein! Over the years they had simply lost that skill. Not only that, but nurses struggled in situations where they were called upon to act quickly based on their own judgement of what needed to be done. Again, this was a hangover from the old Soviet Union days where nurses were trained to deliver patient care strictly according to physicians’ instructions.

During the pandemic when decisions had to be taken quickly and doctors were often occupied elsewhere, this was a problem. So, at the beginning of 2020, a group of colleagues and I decided to change the way we worked. We had just come back from best practice sharing trips in Switzerland and Lithuania where we had seen how much scope was accorded to nurses and the level of autonomy they enjoyed. We also saw how much emphasis was put on patient-centred care. Coming home again we were struck by the limitations of our methods, which were made all the more apparent because of the pandemic. When old-style treatment nurses entered the red zone (COVID-19 treatment and resuscitation areas marked off with red tape on the floor), they simply did not know what to do or how to go about it. The shortcomings of their training were so glaring that we quickly realized that we urgently needed to overhaul the system. So, we developed a new approach which we call the Universal Nurse model.

*Q: Can you explain what that is?*

A: It is basically a systematic approach that encourages nurses to think and act for themselves, independently assessing the condition of the patients, making a nursing diagnosis, developing and implementing a plan in response and evaluating the results.

*Q: What is meant by a nursing diagnosis?*

A: It boils down to the identification of a problem and an associated response. For instance, if the nurse sees that the patient has high blood pressure, she or he follows an algorithm to perform the actions needed to bring it down, for example by calming the patient or administering treatment. The nurse then evaluates the patient’s condition to see if the response has been effective. It may sound like a simple thing to implement, but it is actually quite challenging. Nurses not only have to think for themselves and can no longer turn to the doctor for instruction, they also have to engage with patients, paying close attention to their condition and needs. Both the timeliness and quality of treatment improves. Usually, such profound changes in practice take years to materialize, but the pandemic really speeded the reform process up. The Ministry of Health gave us the green light and we launched a Universal Nurse pilot project a few months after the pandemic started. Typically, the nurses in the pilot are responsible for between 8–12 patients on a general unit and three patients in intensive care, compared to 20–40 patients per the old function-oriented model. To emphasize patient-centred care, the nurses manage the patients assigned to them from the moment they are admitted to the moment they get discharged. The pilot was introduced in three hospitals, with implementation supported by the health ministry and USAID’s (United States Agency for International Development) Local Health System Sustainability Project which focuses on integrated health system strengthening. It’s too early to measure the full impact of the pilot on patient outcomes, but we have early indications of improved patient outcomes and hope to extend the project to the entire republic from the second half of 2022.

*Q: What are the implications of the Universal Nurse approach for staffing levels?*

A: Clearly, a reduction in the number of patients per nurse calls for increased staffing, but, fortunately, staffing levels have remained fairly robust in recent years with around 96–97% of advertised positions being filled. However, there is an important imbalance between staffing levels in urban and rural areas. Unlike many countries we tend to have a surplus of nurses in rural areas where staffing levels can go as high as 120% of the requirement, while in the capital, Bishkek, there are severe staff shortages.

“The pandemic really speeded the reform process up.”

*Q: Why is that?*

A: The main challenge to recruitment is the high cost of living in the city. You need at least US$ 400–500 a month to survive in Bishkek, but young nurses start on less than $100 per month, while experienced nurses with different allowances can earn up to $250. So, if you do not own a flat or have additional sources of income, you can’t make ends meet. In rural areas the situation is exactly the opposite. There, people live off the land and provide for almost everything themselves. In the countryside even a small salary is seen as an important source of income – and a nurse can support a whole family. And if you live up in the mountains – which is nearly always the case in Kyrgyzstan – you also get a 50% salary bonus.

*Q: How are you addressing the situation?*

A: We are trying to secure some financial allowances, at least for nurses operating in the most gruelling conditions such as oncology and intensive care. But our lifeline at the moment is the system of postgraduate work assignments under which it is mandatory for students of state-sponsored medical colleges to work for two years at institutions they are assigned to. To retain them beyond that, we are trying to find ways to secure some kind of bonus for working in cities but so far progress is very limited. In Bishkek, the mayor’s office is paying a little extra, but this is an exception rather than the rule. However, it is important to recognize that the salary is only one part of the equation. I believe that we can do more to attract people into the profession and encourage experienced nurses to stay by expanding the scope of the work and making it more interesting under the Universal Nurse model. We need to transform nurses from simple assistants into true respected professionals with a wide range of duties. I think this may also bring more men into the profession. The gender imbalance in nursing is seen in most countries, and here it’s no different. According to a recent report, women make up 96% of all nurses here. Even in departments where physical strength is really needed, such as in intensive care units or accident and emergency departments, most tasks are still performed by women. So, we are currently working with the nursing association to get more men to join the profession. One of the main obstacles hampering our efforts is that the professional term for nurse in Russian is ‘sister of mercy’, which makes many men believe that this is a purely female profession. It is not. And we need men on our wards. We have even introduced the term ‘brother of mercy’ in all our official and regulatory documents and keep our fingers crossed that the gender balance will improve sooner rather than later.

